# Aging and Decision Making: Socio-Emotional and Contextual Factors

**DOI:** 10.1093/geroni/igaa057.1830

**Published:** 2020-12-16

**Authors:** JoNell Strough, Corinna Loeckenhoff, Susan Charles

## Abstract

Maintaining sound decision-making skills in later life is a key concern in the face of population aging. The four presentations in this symposium highlight the importance of considering socio-emotional and contextual factors when investigating adult age differences in decision making. Together, they show that features of decision contexts such as the way information is presented, along with social relationships and emotional responses, have distinct implications for understanding age effects in decision-related processing and outcomes. Drawing from fuzzy trace theory, Nolte, Löckenhoff and Reyna showed that gist-based (“good,” “extremely poor”) versus verbatim information (exact numbers) was differentially appealing to younger and older adults, with older adults seeking more gist information than verbatim information. Young and Mikels investigated older and younger adults’ integral emotional responses to a behavioral risk-taking task. Younger adults experienced more anger and less contentment than older adults. These emotions differentially predicted risk taking in the two groups. Seaman, Christensen, Senn, Cooper, and Cassidy found age differences in learning about the trustworthiness of social partners. Older adults showed less learning relative to younger adults and invested less with trustworthy partners and more with untrustworthy partners. Smith, Strough, Parker and Bruine de Bruin found that older age, perceiving better decision-making ability than age peers, and perceiving declines in ability over time, were associated with lesser preferences for making decisions with others. In her discussion, Charles will integrate these findings with existing research on aging and decision making and offers directions for future research.

